# Generation of synthetic ground glass nodules using generative adversarial networks (GANs)

**DOI:** 10.1186/s41747-022-00311-y

**Published:** 2022-11-30

**Authors:** Zhixiang Wang, Zhen Zhang, Ying Feng, Lizza E. L. Hendriks, Razvan L. Miclea, Hester Gietema, Janna Schoenmaekers, Andre Dekker, Leonard Wee, Alberto Traverso

**Affiliations:** 1 Department of Radiation Oncology (Maastro), GROW School for Oncology and Reproduction, Maastricht University Medical Centre+, Maastricht, The Netherlands; 2 Department of Radiation Oncology, Key Laboratory of Cancer Prevention and Therapy, Tianjin Medical University Cancer Institute and Hospital, National Clinical Research Center for Cancer, Tianjin’s Clinical Research Center for Cancer, Tianjin, China; 3 Department of Ultrasound, Beijing Friendship Hospital, Capital Medical University, Beijing, China; 4 Department of Obstetrics and Gynecology, GROW-School for Oncology and Developmental Biology, Maastricht University Medical Centre, Maastricht, The Netherlands; 5 Department of Pulmonary Diseases, GROW School for Oncology and Reproduction, Maastricht University Medical Center+, Maastricht, The Netherlands; 6 Department of Radiology and Nuclear Medicine, Maastricht University Medical Centre+, Maastricht, The Netherlands

**Keywords:** Deep learning, Tomography (x-ray computed), Lung, Neural networks (computer), Solitary pulmonary nodule

## Abstract

**Background:**

Data shortage is a common challenge in developing computer-aided diagnosis systems. We developed a generative adversarial network (GAN) model to generate synthetic lung lesions mimicking ground glass nodules (GGNs).

**Methods:**

We used 216 computed tomography images with 340 GGNs from the Lung Image Database Consortium and Image Database Resource Initiative database. A GAN model retrieving information from the whole image and the GGN region was built. The generated samples were evaluated with visual Turing test performed by four experienced radiologists or pulmonologists. Radiomic features were compared between real and synthetic nodules. Performances were evaluated by area under the curve (AUC) at receiver operating characteristic analysis. In addition, we trained a classification model (ResNet) to investigate whether the synthetic GGNs can improve the performances algorithm and how performances changed as a function of labelled data used in training.

**Results:**

Of 51 synthetic GGNs, 19 (37%) were classified as real by clinicians. Of 93 radiomic features, 58 (62.4%) showed no significant difference between synthetic and real GGNs (*p* ≥ 0.052). The discrimination performances of physicians (AUC 0.68) and radiomics (AUC 0.66) were similar, with no-significantly different (*p* = 0.23), but clinicians achieved a better accuracy (AUC 0.74) than radiomics (AUC 0.62) (*p* < 0.001). The classification model trained on datasets with synthetic data performed better than models without the addition of synthetic data.

**Conclusions:**

GAN has promising potential for generating GGNs. Through similar AUC, clinicians achieved better ability to diagnose whether the data is synthetic than radiomics.

## Key points

- We propose a technique that can generate synthetic ground glass opacities.
- Some of the generated images were assessed as real by physicians and imaging quantitative method (radiomics).
- The synthetic data can improve the performance of deep learning classification models.

## Background

Artificial intelligence is a rapidly developing field including many applications in computer vision, such as deep learning (DL) and machine learning methods for the segmentation [1] and the classification [2] of anatomical structures and abnormalities in standard of care diagnostic imaging. A strong effort is dedicated to the implementation of these methods as computer-aided diagnosis (CAD) tools to reduce the time burden of clinical tasks and improve radiologists’ detection accuracy. For lung cancer screening, the number of CAD systems to automatically identify the presence of pulmonary nodules has exponentially increased in the last 10 years. DL methods have shown an increased detection accuracy for all the types of pulmonary nodules (solid, part solid, ground glass opacities) compared to traditional machine learning methods in low-dose screening computed tomography (CT) scans [3, 4].

The success of developing robust and widely applicable deep learning-based CAD systems relies on the availability of a large amount of curated and annotated data. However, annotating data consistently has a cost and is dependent on radiologists’ time and availability. Even when large amount of data is collected for training DL networks, the problem of class imbalance may exist. The class imbalance problem refers to some labels (classes) being more frequent than others. Due to this unbalance, the DL network will learn better how to classify the more frequent samples, with degraded performances for the minority class(es) [5]. In the specific case of pulmonary nodule detection, ground glass nodules (GGN), although accounting for only 2.7 to 4.4% of all nodules, are malignant in 63% of the cases [6].

Next to classical statistical methods such as SMOTE (synthetic minority oversampling technique), researchers have investigated more advanced methods for generating synthetic samples of original data, to increase and balance the original sample size of the training dataset. Recently, generative adversarial networks (GANs) have been proposed as a method to generate synthetic images to improve the existing oversampling techniques [7]. GANs, which are DL algorithms based on game theory, have been applied to several computer vision tasks such as image denoising, reconstruction, and, as mentioned, synthetic data generation [8, 9]. Briefly, GANs consists of two competing actors: a generator and a discriminator. They are used to generate synthetic images/samples and “judge” the quality of the generated images, respectively. The equilibrium is reached when the synthetic (*i.e.,* fake) samples cannot be distinguished from the real distribution [10].

While many studies demonstrated the potential of GANs to generate synthetic images, the generated images/samples have not been evaluated by radiologists, and this limits the acceptance and use of GANs in a clinical setting. In fact, generated images/samples should be representative of the “real” population. However, by only focusing on evaluating at the “human-level” appropriateness of synthetic samples, it is not possible to draw any conclusion whether the introduction of synthetic samples in the training samples will improve the detection performances of CAD systems. In principle, it is expected that adding as many synthetic samples as possible to the original data will lead to a CAD system with better detection performances. It is important to notice that generating synthetic samples via GAN is in itself a learning procedure, where the original data is used to train the networks to generate the synthetic samples. The ratio between original data available and the quality of generated samples is not clear yet.

In this study, we investigated the following research questions:

i. Is it possible to use a GAN model to generate synthetic GGNs on low-dose screening CT scans that are undisguisable by clinicians from the real samples?
ii. How much labelled data is needed to generate synthetic GGNs of sufficient quality to train a CAD for pulmonary nodule detection achieving the same level of performance of a large amount of labelled data?

To answer these questions, we developed an optimised GAN model with dual discriminators to generate GGNs.

## Methods

### Study population

A total of 216 subjects were selected from The Lung Image Database Consortium and Image Database Resource Initiative (LIDC-IDRI) database for this study [11]. In this database, the nodules were classified into five grades by four radiologists: 1 = ground glass opacity (GGO^1^); 2 = intermediate between 2 and 3; 3 = part solid; 4 = intermediate between 4 and 5; 5 = solid. We chose 340 GGN nodules of grades 1 or 2 that were annotated by at least two radiologists for our study. To ensure data quality, further confirmation was performed by a radiologist (author Z.Z.), with 5 years of experience in lung CT, to verify that all the nodules were GGNs.

### Image preprocessing

**Fig. 1:**
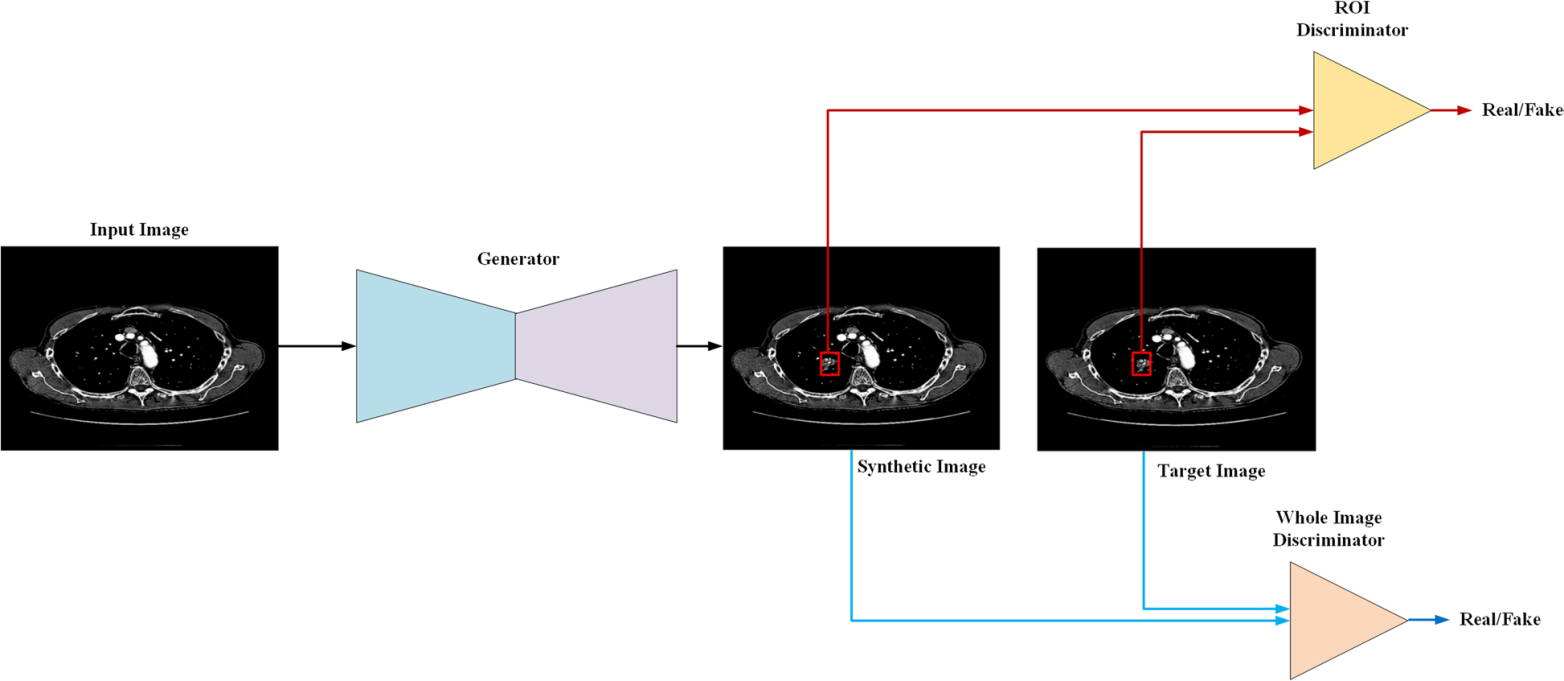
The pipeline for training the model. First, the generator synthetises ground glass nodules from the background according to the input image. Second, the region of interest (ROI) discriminator (red line) and the whole image discriminator (blue line) extract features from the ROI and whole image to classify the synthetic image and the target whether the synthetic image is real

**Fig. 2:**
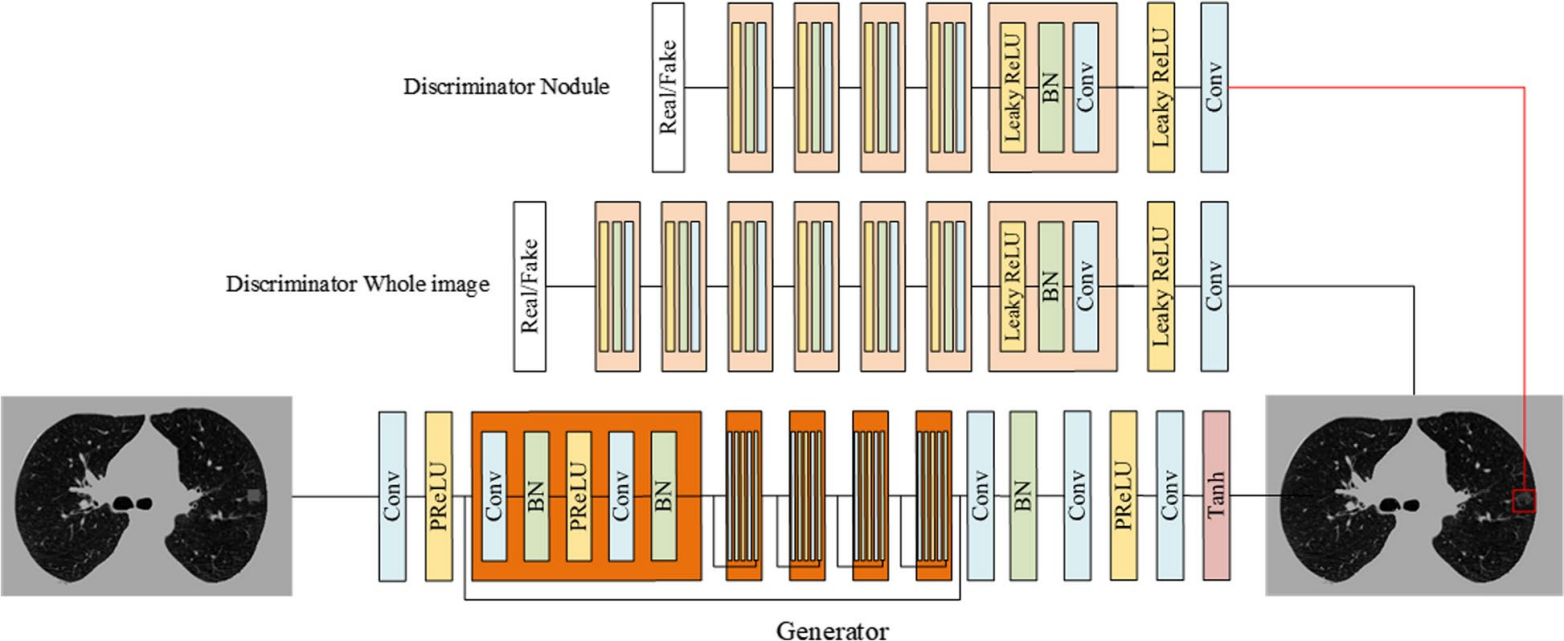
The structure of the network. The generator creates the synthetic ground glass nodule at the position where the mask in the input. The generator is composed of convolutional layers with a kernel size of 3 × 3, the batch normalisation, and the “parametric rectified linear unit” (PReLU) activation function. The discriminator was composed of convolutional layers with a kernel size of 3 × 3, the batch normalisation, and the leaky PReLU activation function

In the preprocessing methods, first, the two-dimensional slices with annotation as GGN from the CT volume were extracted. Second, in order to avoid interference from external tissues of the lung, we first cropped the lungs from the tissue and background with a seed-filling algorithm, which starts from an inner point of the polygon area and draws points with the given grey level from inside to outside until the boundary is found. Third, the cropped images were padded by 0 in the background to keep every image having the same sizes (512 × 512) in the dataset. Fourth, we normalised the data to the range 0–1, as is the standard practice in computer vision. Fifth, we erased the nodules from the original position and saved them as region of interest (ROI) for the training set. In general, each training batch contained two images: the original image as the target image, which serves as the ground truth for the generator (as shown in Figs. 1 and 2), and another image is the input image, in which stripped the nodule area, *i.e.,* the ROI region was processed as blank for the input image. As shown in Fig. 1, the network generates the nodule from the input image. In addition, after generation, there are two discriminators (whole image discriminator and ROI discriminator) to evaluate the quality of the whole image and the ROI where the nodule is.

### Construction of the DL model

The super-resolution generative adversarial network (SRGAN) was used as the backbone of the generator [12]. SRGAN compares the features difference in the model between a pair of data and train the discriminators to improve the realism of the recovered images. Both the whole image discriminator and ROI discriminator are based on a ResNet [13] which is a widely used classical classification networks combined by residual blocks with different input sizes and depths of the network. The structure of the network is shown in Fig. 2. For training the network, the loss function was as follows:

1 $${L}_{D2SRGAN}={({L}_{ssim}+{L}_{adversarial})}_{whole image}+{({L}_{ssim}+{L}_{adversarial})}_{ROI image}$$

2 $${L}_{adversarial}=\sum_{n=1}^{N}-logD(G(x))$$

3 $${L}_{ssim}(x,y)=1-\frac{(2{\mu }_{x}{\mu }_{y}+{C}_{1})+({\sigma }_{xy}+{C}_{2})}{({{\mu }_{x}}^{2}{{\mu }_{y}}^{2}+{C}_{1})({{\sigma }_{x}}^{2}{{\sigma }_{y}}^{2}+{C}_{2})}$$

The ${L}_{ssim}$ can be used to compare the similarity between two images. In this loss function, the whole image is separated into two parts to calculate the loss function respective. G and D represent the generator and discriminator, *x* is the input of the generator. ${\mu }_{x}$ and ${\mu }_{y}$ represent the average of input *x*,*y* respectively. ${\sigma }_{x}$ and ${\sigma }_{y}$ represent the standard deviation of input *x*,*y* respectively. ${\sigma }_{xy}$ is the covariance of *x* and *y*. C1 and C2 are constants to avoid system errors caused by the denominator being zero.

All images were loaded with an unchanged original size of 512 × 512. The input size of the discriminator for the whole image and the ROI image were 512 × 512 and 32 × 32, respectively. An Adam optimizer was used to train both the generator and the discriminator with a learning rate of 0.0001. This model was trained using an NVIDIA Tesla V100 SXM2 32 GB graphics processing unit.

### Evaluation of model performance

We evaluated the model performance using both subjective (visual Turing test, VTT) and objective (radiomics) approaches. VTT is an assessment method that evaluation the ability of a human or doctors to identify attributes and relationships from images [14]. Subjective evaluations were performed by two radiologists (authors R.M. and H.G.) and two pulmonologists (authors L.H. and J.S.), who all had more than 5 years of experience in lung CT imaging and on a daily basis evaluate chest CT scans. One hundred images (50 real and 50 synthetic GGNs) were divided into four batches and converted to a DICOM (Digital Imaging and COmmunications in Medicine) file with 25 slices of images, and each physician was randomly assigned to one of these batches. The physicians categorised the real and synthetic GGNs into four classes based on this categorical scale: confidently fake, leaning fake, leaning real, and confidently real.

To perform an objective evaluation, radiomic features were calculated from the original and generated data. Radiomics refers to the extraction of quantitative information from medical images by computing the statistical, morphological, and texture features. The following feature categories were extracted using the open source Pyradiomics package (version 3.0.1) with default values: first order statistics (*n* = 18), grey level co-occurrence matrix (*n* = 24), grey level dependence matrix (*n* = 14), grey level run length matrix (*n* = 16), grey level size zone matrix (*n* = 16), and neighbouring grey tone difference matrix (*n* = 5) [15–17].

The Kolmogorov–Smirnov test was used for the analysis of whether the distribution of radiomics features were similar between the real and synthetic GGNs. We considered significant *p* values lower than 0.05.

The results of the subjective and objective evaluations were analysed using the area under the curve (AUC) at receiver operating characteristic analysis. For the subjective evaluation, we took into consideration the VTT results. For the objective evaluation, to compare the classification ability of radiomics and radiologist, a logistic regression model was build based on radiomic features to classify both real and synthetic GGNs. The same dataset was used for the physician evaluations and the radiomics logistic regression model, with a fourfold cross-validation.

In addition, we also investigated whether the synthetic GGNs can improve the performance of a CAD algorithm trained for recognising GGNs from all types of nodules in the LIDC-IDRI dataset and how the performance changed as a function of labelled data used in the training.

As a CAD, we used a basic ResNet as the DL classification network with a cross-entropy loss function. First, we separated the dataset into 10 training subsets and an independent test set. We trained the classification network on portions of the original data ranging from 10 to 100% of the real data and we separately inferred on the test set. Then, we trained the classification network on the original data added systematic data generated by the GAN network trained in 10% to 100% of real data.

## Results

**Fig. 3:**
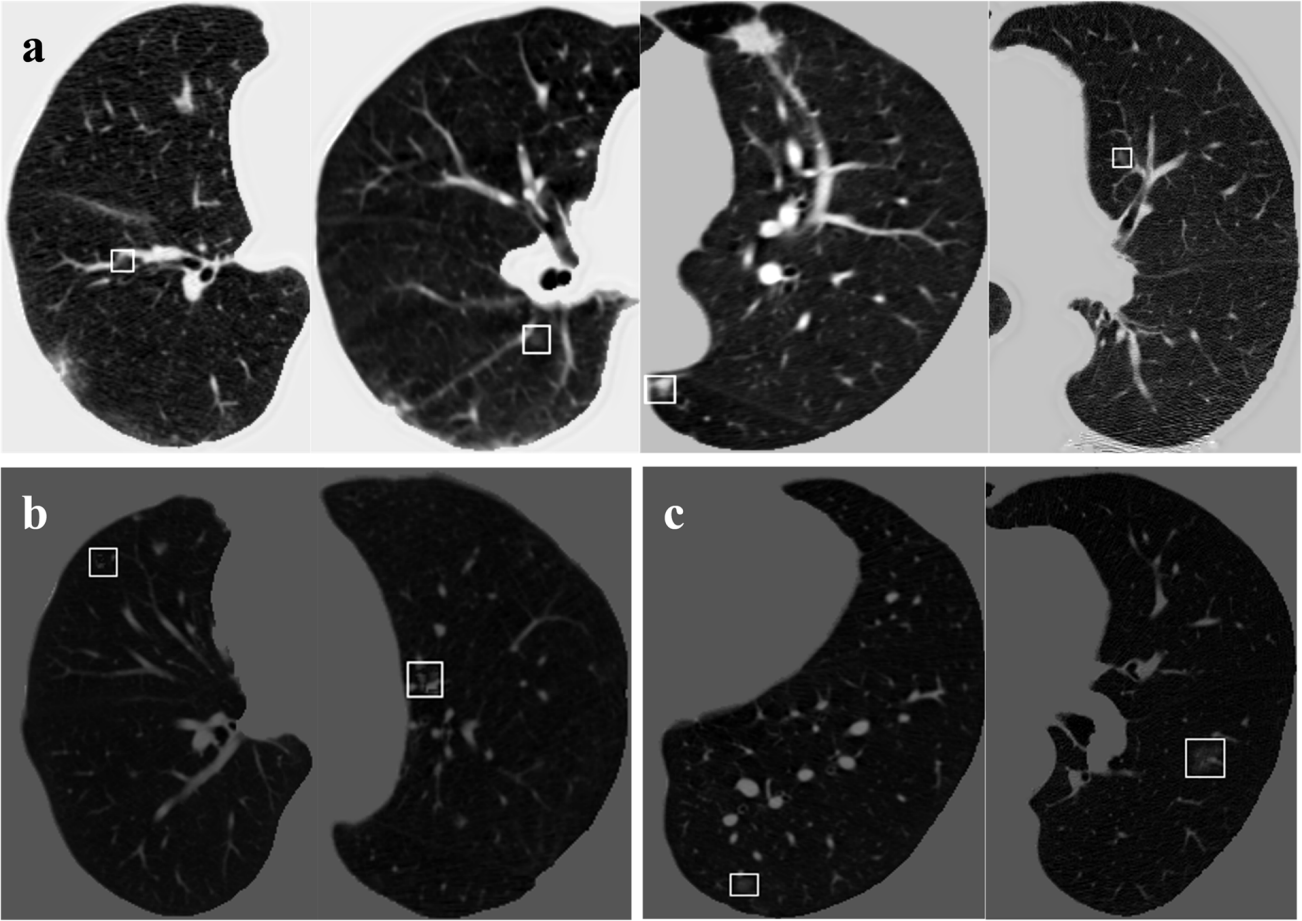
Examples of synthetic ground glass nodules (GGNs), the GGNs were categorised by physicians to four categories: confidently fake, leaning fake, leaning real, and confidently real. **a** Synthetic GGNs classified as “real” by clinicians. **b** Synthetic GGNs with less convincing generated lesions (classified as “leaning fake”). **c** A real GGNs in the original LIDC-IDRI dataset

Examples of synthetic GGNs generated in different parts of the lungs with different surrounding tissues are shown in Fig. 3. Nodules classified as fake (Fig. 3b) show more unnatural characteristics in terms of intensity and morphology than nodules classified as “real” (Fig. 3a); specifically, “fake nodules” have very high fixed grey values and regular shapes such as rectangles.

### VTT results

**Fig. 4:**
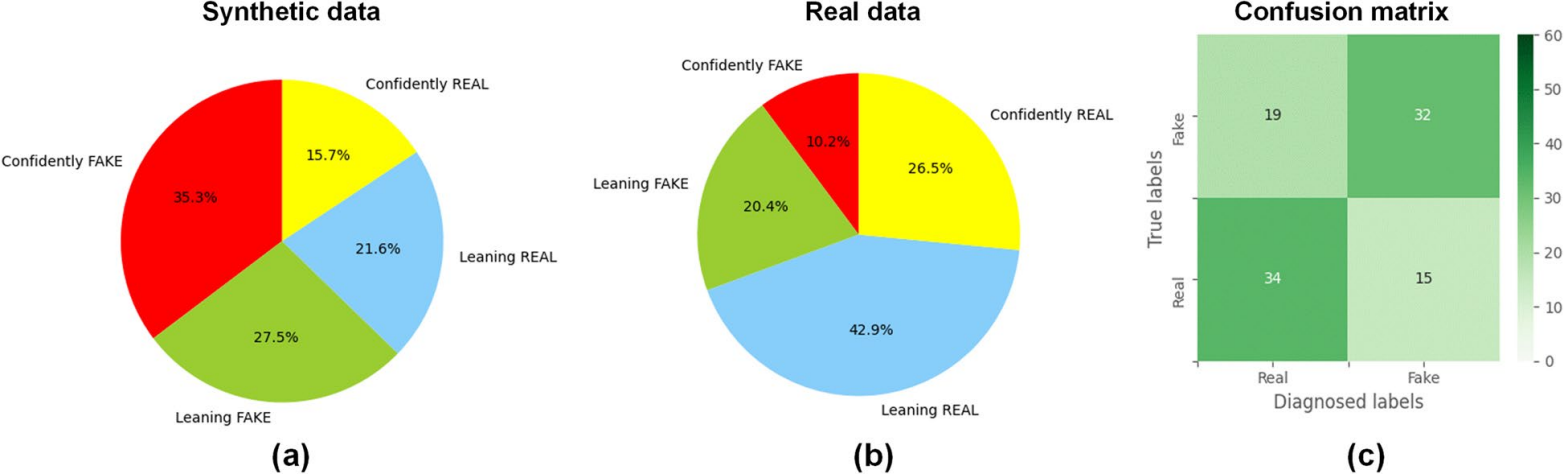
Visual Turing test results. **a**, **b** Prediction distribution in synthetic and real ground glass nodules. **c** Confusion matrix for the prediction

Figure 4 presents the combination of the classification results for the four clinicians: of 51 synthetic GGNs, 19 (37%) were classified as real by clinicians, 8/51 (16%) were classified as confidently real, and 11/51 (22%) were classified as leaning real.

### Radiomics

**Fig. 5:**
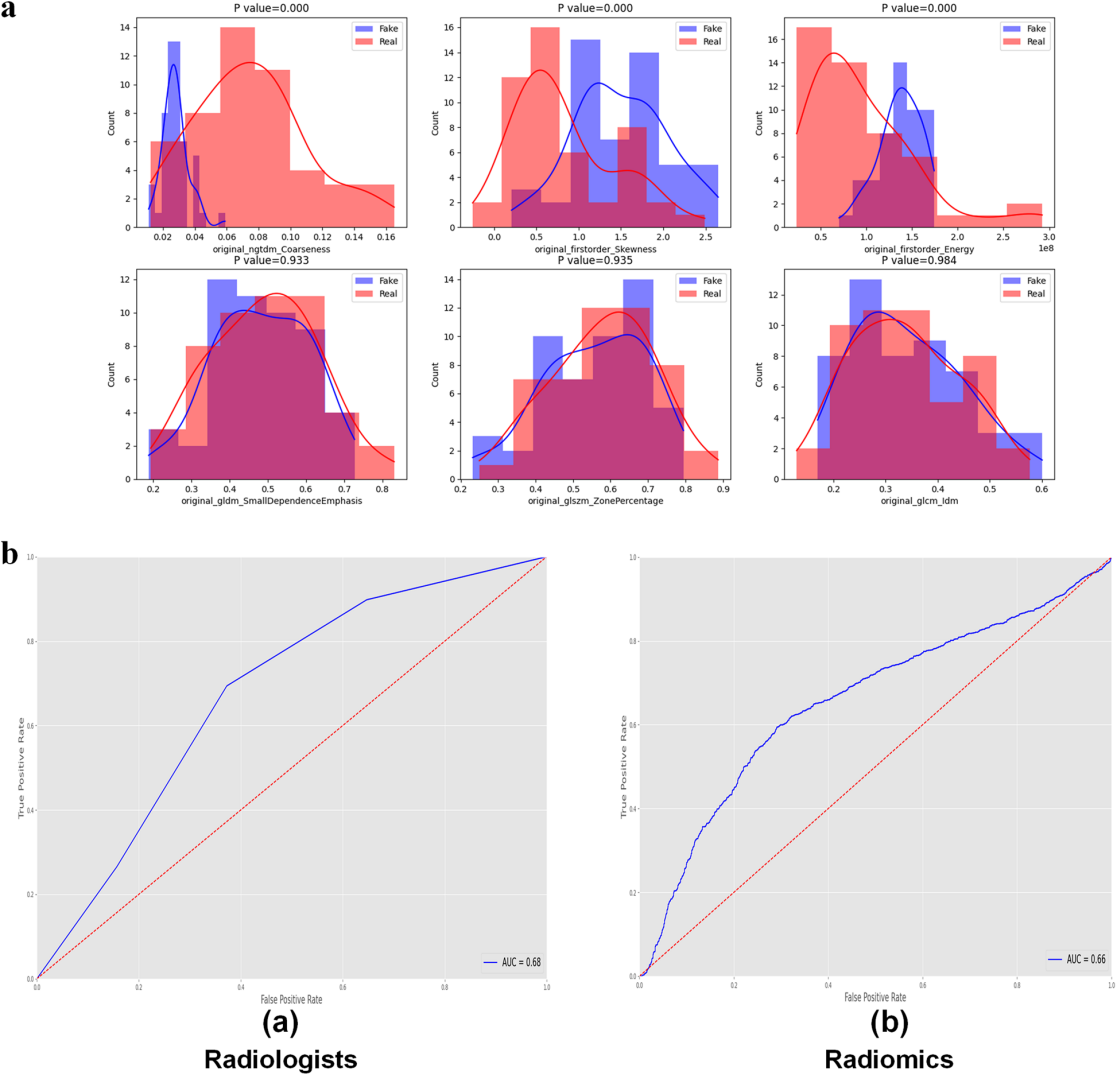
**a** Examples for the comparison of radiomics features distribution between real and fake ground glass nodules (GGNs). The comparison of radiomics features distribution extracted from synthetic and real images with minimum three *p*-values shows in the upper row. The comparison of radiomics features distribution extracted from synthetic and real images with maximum three *p*-values shows in the lower row. **b**, **c** Receiver operating characteristic curve of the prediction of distinguishing real and fake GGNs. by radiologists (**a**) and by the logistic regression model (**b**)

Of a total of 93 features, 58 (62.4%) showed no significant difference (*p* ≥ 0.052) between synthetic and real GGNs, and the detailed results are provided in Table 1. Figure 5a shows the comparison of the distribution of radiomic features between real and synthetic GGNs, the histogram shows the counts of specific feature values, and the differences (*p*-values) in the extracted radiomic features between real and synthetic GGNs were calculated. The receiver operating characteristic curves constructed based on the results of VVT by clinicians and logistic regression model developed by radiomics features are shown in Fig. 5b. We observed a similar classification performance of clinicians (0.68) and radiomics (0.66), with no-significantly different (*p* = 0.23). However, the clinicians achieve significant great performance accuracy around 0.74, better than the 0.62 radiomics accuracy (*p* < 0.001). The clinicians achieves better ability to diagnosis whether the data is synthetic than radiomics.

**Table 1:** Comparison between real and deep learning-generated radiomic features (*p*-values according to the Kolmogorov–Smirnov test)

Class	Feature name	*p*-value
Grey level co-occurrence matrix (GLCM)	Inverse difference moment	0.984025
Grey level size zone matrix (GLSZM)	Zone percentage	0.934856
Grey level dependence matrix (GLDM)	Small dependence emphasis	0.932657
Grey level co-occurrence matrix (GLCM)	Inverse difference	0.926064
First order	Robust mean absolute deviation	0.903346
GLSZM	Small area low grey level emphasis	0.860311
Grey level run length matrix (GLRLM)	Run percentage	0.827381
GLRLM	High grey level run emphasis	0.729491
GLSZM	Grey level non-uniformity normalised	0.696774
GLRLM	Long run emphasis	0.676057
GLCM	Sum entropy	0.658063
GLRLM	Long run high grey level emphasis	0.652292
GLRLM	Run entropy	0.652292
First order	Entropy	0.643479
GLCM	Inverse variance	0.616719
GLRLM	Short run high grey level emphasis	0.582172
GLDM	High grey level emphasis	0.574195
GLCM	Joint energy	0.570327
GLCM	Joint entropy	0.570327
GLRLM	Run length non-uniformity normalised	0.570327
GLRLM	Short run emphasis	0.570327
First order	90 percentile	0.541180
GLDM	Small dependence low grey level emphasis	0.512551
First order	Interquartile range	0.498064
GLCM	Inverse difference normalised	0.456086
GLDM	Large dependence emphasis	0.450880
GLDM	Dependence variance	0.445137
GLSZM	Low grey level zone emphasis	0.445137
First order	Mean absolute deviation	0.414534
GLCM	Autocorrelation	0.407415
GLDM	Dependence non-uniformity normalised	0.403944
First order	Mean	0.389392
GLRLM	Run variance	0.375333
GLRLM	Grey level non-uniformity normalised	0.324190
GLCM	Maximum probability	0.307686
Neighbouring grey tone difference matrix (NGTDM)	Strength	0.272504
GLCM	Cluster tendency	0.267111
GLCM	Inverse difference moment normalised	0.264157
GLDM	Dependence entropy	0.261878
GLRLM	Short run low grey level emphasis	0.227646
First order	Minimum	0.212067
GLSZM	Large area high grey level emphasis	0.202291
First order	Root mean squared	0.186989
GLSZM	Large area emphasis	0.178996
GLDM	Grey level variance	0.170028
GLCM	Joint average	0.160908
GLCM	Sum average	0.160908
First order	Uniformity	0.133892
GLDM	Small dependence high grey level emphasis	0.124894
GLSZM	Zone variance	0.119210
First order	Variance	0.108119
GLCM	Sum squares	0.108119
GLSZM	High grey level zone emphasis	0.105973
GLDM	Large dependence low grey level emphasis	0.082337
GLSZM	Size zone non-uniformity normalised	0.074667
GLSZM	Small area emphasis	0.073186
GLSZM	Large area low grey level emphasis	0.069577
GLRLM	Grey level variance	0.066007
GLCM	Informational measure of correlation 2	0.052283
GLRLM	Low grey level run emphasis	0.045409
GLSZM	Small area high grey level emphasis	0.044462
GLCM	Cluster prominence	0.022046
GLSZM	Grey level variance	0.021275
NGTDM	Contrast	0.020502
First order	10^th^ percentile	0.015568
GLDM	Low grey level emphasis	0.014150
GLCM	Difference entropy	0.011605
GLSZM	Zone entropy	0.010051
GLRLM	Long run low grey level emphasis	0.008825
GLCM	Informational measure of correlation 1	0.006491
GLCM	Difference average	0.005938
GLCM	Maximal correlation coefficient	0.005586
GLDM	Large dependence high grey level emphasis	0.003520
First order	Maximum	0.002755
GLCM	Cluster shade	0.002638
First order	Range	0.001136
First order	Median	0.000355
GLCM	Contrast	0.000251
GLDM	Dependence non-uniformity	0.000230
GLSZM	Size zone non-uniformity	7.60E-05
NGTDM	Busyness	6.60E-05
GLCM	Correlation	2.40E-05
GLSZM	Grey level non-uniformity	1.40E-05
NGTDM	Complexity	1.40E-05
GLCM	Difference variance	5.00E-06
NGTDM	Coarseness	0.000000
First order	Skewness	0.000000
First order	Energy	0.000000
First order	Total energy	0.000000
First order	Kurtosis	0.000000
GLRLM	Run length non-uniformity	0.000000
GLDM	Grey level non-uniformity	0.000000
GLRLM	Grey level non-uniformity	0.000000

### DL classification network

**Fig. 6:**
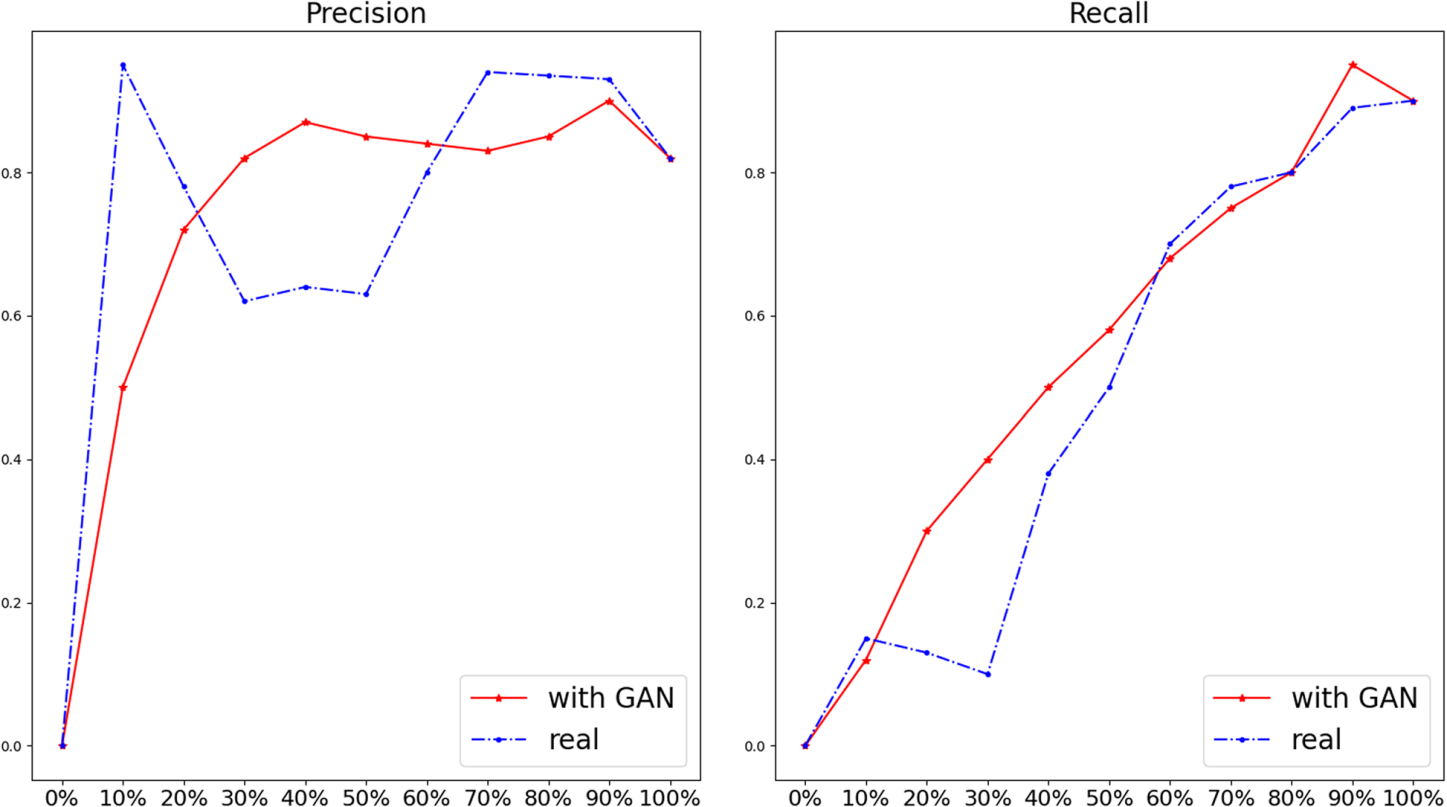
Comparison precision (*i.e.,* positive predictive value) and recall (*i.e.,* sensitivity) between real and added synthetic dataset in different percentages of the training set. The blue and the red lines present the performance of the deep learning classification model trained by real data and the real data plus synthetic data, respectively. The horizontal axis label is the percentage of training data in the dataset. The vertical axis label is the score of precision and the recall with the range from 0 to 1

The results of the DL classification network trained using decreasing portions of the dataset are shown in Fig. 6. When the dataset is 90%, the precision (*i.e.,* positive predictive value) was similar between the two groups. However, when the dataset decreased to 50%, the performance of the real data only group significantly decreased. On the other hand, synthetic GGNs can increase precision in training the DL network. When the sample decreased to 10%, the real data has better performance than by adding synthetic data. From Fig. 6b, the recall (*i.e.,* sensitivity) of GGN was decreasing when decreasing the dataset both in real data only and real data with GAN groups. However, in most cases, models trained on datasets with synthetic data performed better than models without the addition of synthetic data.

## Discussion

In the present study, we applied a GAN-based model with double discriminators to generate GGN in low-dose CT scans. We benchmarked the performance of the model using a qualitative (VTT with clinicians) and a quantitative approach (radiomics).

To our knowledge, only one previous study proposed the use of GANs to generate lung lesions and performed a VTT [18], which showed that 67% and 100% of the fake nodules were marked as real by two radiologists, respectively. Differences exist between this study and our study: in the VVT of the cited study [16], the radiologists reviewed the generated lesions, but the surrounding tissues or the entire lungs were not included in the field of view. Moreover, the surrounding tissues and the lung background that has relationship with nodules were not considered when training and generating the nodules. Conversely, we generated GGNs from the whole lung to use the anatomical dependence with the background tissue [19]. However, the relatively small size of our study compared to the previous research [18] probably influenced the results of the visual Turing test.

Based on our VTT evaluation, we have shown that GAN-generated lung lesions have the potential to be very consistent with real lesions. This gives us the opportunity to use GAN-generated data to solve real-world problems, such as using the generated data to train and test junior doctors, especially for hospitals that do not have large cohort datasets, long-time established picture archiving and communication systems, as privacy-preserving synthetic open datasets for research purposes.

More than half of the radiomic features were not statistically different between DL-generated and real nodules, proving that the generated GGNs are acquiring or learning detailed features from the real sample. Furthermore, these consistent radiomic features cover all classes, which could support the conclusion that the proposed approach mimics different aspects of real nodules. Conversely, one third of the features in this study showed significant differences in the distribution between the generated and real GGNs. Based on the radiomics results and the clinicians’ opinion, we think that the low complexity of the generated GGNs is the main reason for the discrepancy between the generated and real GGNs. For example, the *p*-value of the radiomic features *coarseness* (which can measure the spatial change rate) and *complexity* (which can measure the non-uniformity of local grey levels) between real and synthetic GGNs are close to 0, supporting our hypothesis. We hypothesise the following explanations: (i) the data source is derived from public databases that have low resolution and lots of noise, and (ii) we did not optimise the training process by specifically including radiomics features in the loss function.

Based on the radiomics results, we built a “radiomics physician” to discriminate between real and generated GGNs, which interestingly is generally consistent with the discriminatory ability of real physicians. It is worth noting that the “radiomics physician” model was trained based on a sample of 100 cases, and the physicians have more than 5 years of experience. Overall, it is a challenging task to discriminate between real and generated GGNs for “radiomics physicians” and real physicians.

Finally, we wanted to test how data augmentation with GAN will affect the detection accuracy of a CAD system. Figure 6 shows that adding synthetic GGNs to the original dataset improves the performance of our DL CAD system. However, there was no significant contribution when the size of the training dataset is under 10% and over 70% of the original sample size. We hypothesise that when the training data is under 10%, there is an insufficient number of samples to train the GAN. A GAN trained on only a few samples cannot synthesise the rich diversity and complexity of real GGNs. Based on the results (Fig. 6), we conclude that the performance of the DL model increases with the sample size in certain ranges of real data samples. However, as shown in Fig. 6, the performance of the DL model cannot be improved after a threshold value larger than the sample size, which is the plateau of the model. Specifically, for effective dataset size to train a GAN, around 50% of training data which include around 100 samples of GGN has the biggest increase in accuracy of the classification model when synthetic GGN are added. Overall, from our experiment, we found that:

i. Synthetic data has the ability to increase the performance of a DL model unless only a few training samples can be used;
ii. From the perspective of cost and effectiveness, around 100 samples are sufficient to develop a GAN model that can generate realistic GGNs to significant improve the performance of the detection GGN model.

This study has some limitations. First, we used a public dataset for training the model, but we want to extend the work to other datasets. In future studies, we will add high-resolution data from our centre for model enhancement. Second, we only focused on GGNs, because of their lower incidence compared to other types of nodules. However, the dimension and density variation of the included GGNs is limited, which has the potential risk of obtaining optimistic radiomic assessment results. We will perform transfer learning to generate lung nodules and tumours in the future based on the model in this study. Furthermore, the diagnosis of malignant GGN is a challenging task for clinical practice. However, in this study, we did not generate benign or malignant GGN. To address this issue, we are collecting data from the real world with follow-up endpoints and trying to generate qualitative GGN, especially malignant GGN.

Third, we generated only two-dimensional samples. However, generating three-dimensional (3D) images is costly for model training, first, because 3D GANs have a larger number of parameters which need more training data and also have a significantly higher requirement in hardware when the input data has large scale such as CT images. In the future work, we will consider the model compression to decrease the requirement of hardware and the size of dataset for training the 3D GAN. We tried to perform our visual Turing tests by getting closer as much as possible to a real clinical scenario. Nevertheless, it was out of the scope of this study to integrate our DL models within the clinical workstations available to our radiologists. As proof-of-concept, we proposed to our radiologists the generated and real pulmonary nodules as two-dimensional axial CT images in the standard lung window. Future work will include the production of the generated nodules in standard DICOM formats in all the 3D projections. We are also investigating the possibility to invest in the development of a cloud-based platform to homogenise visual Turing tests for similar experiments. In addition, we did not evaluate the morphological features between the generated and real GGNs.

Fourth, we have not discussed the trend of data requirement for different task, such as what happens when the quality of data is decreased, how many data points need to be added when the target size us increased, and whether different sources such as CT and magnetic resonance imaging influence the dataset requirements. In the future work, we will design experiments to figure out the connection between the data requirement and different tasks.

Fifth, according to the results of the radiomics part, there are still considerable differences between the real and generated GGO, and more than one third of the radiomic feature values were different, which may be a reflection that the GAN method proposed in this study is not optimal. Based on this result, there is still much potential for improvement of our algorithm, with a particular focus on improving the level of complexity of the textures.

Sixth, we did not conduct interobserver and intraobserver testing and the degree of disagreement between different readers was not assessed. On the other hand, in our experience, the differences between the readers (physicians) included in this study were limited to the same broad category, *i.e.,* real or fake. For example, nodules labelled as “confidently real” by one physician have the possibility of being labelled as “leaning real” instead of “confidently/leaning fake” by other physicians.

Finally, despite the GANs are an elegant data generation mechanism gaining more and more popularity in the medical field, most of them still present a high level of complexity compared for example to traditional DL algorithms such as convolutional neural networks. For example, there is no consensus on the most appropriate metric to be used to stop the training at the best point (global minimum of the loss function). This will sometimes lead to a not satisfactory quality of the generated data. Especially when dealing with medical images, the risk of introducing novel, undesired artefacts, and blurry images is not negligible.

In conclusion, in this study, we used GANs to generate GGN and validated these by four physicians and radiomics approaches, showing that GAN methods have great potential for augmentation of the original dataset.

## Data Availability

The datasets applied during the current study are available in the https://wiki.cancerimagingarchive.net/display/Public/LIDC-IDRI.

## Abbreviations

3D: Three-dimensional
AUC: Area under the curve
CAD: Computer-aided diagnosis
CT: Computed tomography
DL: Deep learning
GGN: Ground glass nodule
GGO: Ground glass opacity
LIDC-IDRI: Lung Image Database Consortium and Image Database Resource Initiative
ROI: Region of interest
SRGAN: Super-resolution generative adversarial network
VTT: Visual Turing test

## Footnotes

1 GGO is defined as a type of GGNs showing a misty increase in lung attenuation without obstructing the underlying vascular markings; GGOs can also be called as “pure GGNs,” *i.e.,* GGNs showing solely a GGO component.
